# Diffuse large B-cell lymphoma: the significance of CD8^+^ tumor-infiltrating lymphocytes exhaustion mediated by TIM3/Galectin-9 pathway

**DOI:** 10.1186/s12967-024-05002-3

**Published:** 2024-02-18

**Authors:** Qiqi Zhu, Yiming Yang, Kexin Chen, Qiaoyu Zhang, Yifan Huang, Shunhai Jian

**Affiliations:** 1 Institute of Basic Medicine and Forensic Medicine, North Sichuan Medical College, Nanchong, 637000 China; 2 Department of Pathology, North Sichuan Medical College, Affiliated Hospital of North Sichuan Medical College, No. 1 Maoyuan Nan Road, Nanchong, 637000 Sichuan China

**Keywords:** Diffuse large B-cell lymphoma, CD8^+^ tumor-infiltrating lymphocytes, TIM3/Galectin-9 pathway, Exhaustion, Immune checkpoint, Single-cell RNA sequencing, Prognosis, Immunotherapy

## Abstract

**Background:**

Overexpression of T-cell immunoglobulin and mucin domain-containing protein 3 (TIM3) is related to the exhaustion of CD8^+^ tumor-infiltrating lymphocytes (TILs) in diffuse large B-cell lymphoma (DLBCL). However, the mechanism of TIM3-mediated CD8^+^TILs exhaustion in DLBCL remains poorly understood. Therefore, we aimed to clarify the potential pathway involved in TIM3-mediated CD8^+^TILs exhaustion and its significance in DLBCL.

**Methods:**

The expression of TIM3 and its correlation with CD8^+^TILs exhaustion, the key ligand of TIM3, and the potential pathway of TIM3-mediated CD8^+^TILs exhaustion in DLBCL were analyzed using single-cell RNA sequencing and validated by RNA sequencing. The biological significance of TIM3-related pathway in DLBCL was investigated based on RNA sequencing, immunohistochemistry, and reverse transcription-quantitative polymerase chain reaction data. Finally, the possible regulatory mechanism of TIM3-related pathway in DLBCL was explored using single-cell RNA sequencing and RNA sequencing.

**Results:**

Our results demonstrated that CD8^+^TILs, especially the terminally exhausted state, were the major clusters that expressed TIM3 in DLBCL. Galectin-9, mainly expressed in M2 macrophages, is the key ligand of TIM3 and can induce the exhaustion of CD8^+^TILs through TIM3/Galectin-9 pathway. Meanwhile, high TIM3/Galectin-9 enrichment is related to immunosuppressive tumor microenvironment, severe clinical manifestations, inferior prognosis, and poor response to CHOP-based chemotherapy, and can predict the clinical efficacy of immune checkpoint blockade therapy in DLBCL. Furthermore, the TIM3/Galectin-9 enrichment in DLBCL may be regulated by the IFN-γ signaling pathway.

**Conclusions:**

Our study highlights that TIM3/Galectin-9 pathway plays a crucial role in CD8^+^TILs exhaustion and the immune escape of DLBCL, which facilitates further functional studies and could provide a theoretical basis for the development of novel immunotherapy in DLBCL.

**Supplementary Information:**

The online version contains supplementary material available at 10.1186/s12967-024-05002-3.

## Introduction

Diffuse large B cell lymphoma (DLBCL), which accounts for 30–40% of all non-Hodgkin lymphomas, is a heterogeneous disease with significant differences in clinical manifestations, pathological subtypes, genetic features, and prognosis among patients [1]. Cyclophosphamide, doxorubicin, vincristine, and prednisone (CHOP)-based chemotherapy is the main treatment of DLBCL, but 40% of patients showed no obvious response [2].

The tumor microenvironment (TME) of DLBCL mainly consists of tumor cells, accompanied by immune cells and matrix components, such as tumor-infiltrating lymphocytes (TILs), macrophages, and natural killer (NK) cells [3, 4]. There is a complex interaction network between the tumor cells and TME, the disturbance of which is an important factor affecting the occurrence and progression of DLBCL [5, 6].

TILs are crucial members of TME in DLBCL, in which CD8^+^TILs are the main components that deliver anti-tumor immune response [7, 8]. We previously analyzed the characteristics of TME in DLBCL by flow cytometry and found that CD8^+^TILs may be an important cause of the heterogeneity in DLBCL [9], and the proportion of CD8^+^TILs and the expression of PD-1 play a pivotal role in prognosis evaluation [10]. The increase of immune checkpoint (IC) in DLBCL may inhibit the immune function by inducing CD8^+^TILs exhaustion, resulting in poor prognosis. These results also indicated that IC blockade (ICB) may help to restore the immune function of exhausted CD8^+^TILs [11–13], and preliminarily revealed the application prospect of ICB therapy in DLBCL. However, only limited patients could benefit from classical ICB therapy such as PD-1/PD-L1 blockade due to the developed resistance [14, 15]. Therefore, it is necessary to explore novel biological indicators to prospectively screen patients who may respond to ICB therapy, and to search for novel IC for monotherapy or combination therapy, to improve the prognosis of DLBCL patients.

TIM3, the member of the TIM family of immunomodulatory proteins encoded by HAVCR2, is mainly expressed in CD8^+^T lymphocytes, CD4^+^T lymphocytes, NK cells, and monocytes [16]. In many tumors such as melanoma [17] and gastric cancer [18], the increase of TIM3 is associated with poor prognosis and CD8^+^TILs exhaustion. Nevertheless, there are contradictory conclusions about the effect of TIM3 expression on the prognosis of DLBCL patients [3, 6, 13, 19, 20]. Previous studies demonstrated that the increase of TIM3 may be related to CD8^+^TILs exhaustion and immune deficiency in DLBCL [13, 21]. TIM3 induces CD8^+^TILs exhaustion and inhibits its anti-tumor function mainly by binding to its ligands (Galectin-9, HMGB1, CEACAM1, and Ptdser) [16]. However, the mechanism of TIM3-mediated CD8^+^TILs exhaustion in DLBCL remains elusive.

In this study, the expression of TIM3 and its correlation with CD8^+^TILs exhaustion, the key ligand of TIM3, and the potential pathway of TIM3-mediated CD8^+^TILs in DLBCL were analyzed. The correlations between TIM3-related pathway and the TME composition, the clinical features, prognosis, and the response to ICB therapy were evaluated. Furthermore, the possible regulatory mechanism of TIM3-related pathway in DLBCL was explored. This study aims to elucidate the potential pathway involved in the TIM3-mediated CD8^+^TILs exhaustion, which could provide a theoretical basis for exploring novel immunotargets of DLBCL.

## Materials and methods

### Patients

De novo DLBCL cases (n = 100) from January 2016 to November 2023 were identified from the Department of Pathology of Affiliated Hospital of North Sichuan Medical College. The diagnosis was provided based on the World Health Organization-classified diagnostic criteria for DLBCL (4th edition, 2018). The exclusion criteria were a recurrent history of DLBCL and secondary DLBCL. Relevant clinical data were obtained by reviewing electronic medical records, and pathological information was obtained by examining pathological materials. Follow-up information was collected in December 2023 through telephone interviews or electronic medical record reviews. Survival time was calculated from the date of pathological diagnosis to the date of death or the last follow-up. This study was approved by the Ethics Committee of North Sichuan Medical College. The committee waived the requirement for informed consent as the data for the patients included in the study were retrospectively analyzed.

### Single-cell RNA sequencing

Single-cell RNA sequencing (scRNA-seq) data of 3 reactive lymph node (rLN) tissue samples and 3 DLBCL lymph node biopsy tissue samples were acquired from the heiDATA database (https://heidata.uni-heidelberg.de) [22]. For quality control, genes with detected expression in less than 0.1% of all the cells, and cells with less than 500 detected genes were removed. Additionally, cells with the percentage of mitochondrial genes (> 10%) and identified as doublets were also excluded. Harmony package was used to correct the batch effect among different samples. Finally, a total of 18,113 cells were included for further analysis based on quality control metrics. After the identification of highly variable genes, the first 15 principal components (PCs) (resolution = 0.6) were applied for Uniform manifold approximation and projection (UMAP) analysis. We used the Seurat package in R (version 4.0.5) to perform data filtering, normalization, principal component analysis (PCA), and UMAP after quality control. The Seurat function ‘FindAllMarkers’ and Wilcoxon test were used to identify marker genes for each cluster. Monocle package was used to perform the cell trajectory analysis of CD8^+^TILs. CellChat package was used to quantify the ligand–receptor interactions to evaluate intercellular communication in DLBCL. Single-Cell rEgulatory Network Inference and Clustering (SCENIC) analysis was used to predict the transcription factors (TFs) in the regulation of TIM3 in CD8^+^TILs.

### RNA sequencing

RNA sequencing data and the available survival information for DLBCL patients were obtained from GSE181063 (n = 1310), GSE10846 (n = 412), and GSE53786 (n = 119) from the Gene Expression Omnibus (GEO) database. Pearson’s correlation coefficients were used to assess the correlation among HAVCR2 and its ligands, CD8A, CD8B, IC, and TFs genes, as well as LGALS9, CD163, CD206/MRC1, IFNG, IFNGR1, and IFNGR2. The Tumor Immune Dysfunction and Exclusion (TIDE) score was calculated through the TIDE software (http://tide.dfci.harvard.edu/) to predict the response to ICB therapy. CIBERSORT (https://cibersort.stanford.edu/) was used to evaluate the proportion of tumor-infiltrating immune cells in the TME of DLBCL, covering T cells, B cells, NK cells, macrophages, dendritic cells (DC), eosinophils, and neutrophils.

### Immunohistochemistry

Immunohistochemistry (IHC) was performed on formalin-fixed, paraffin-embedded (FFPE) lymph node tissue sections of DLBCL (n = 100) using anti-TIM3 (Abcam, EPR22241) and anti-Galectin-9 (Cell Signaling, D9R4A). The stains were manually evaluated as the average counts of TIM3^+^ TILs per high power field (HPF) in the hotspot, and the average percent of Galectin-9^+^ cells.

### Reverse transcription-quantitative polymerase chain reaction

The total RNA was extracted from FFPE lymph node tissue sections of DLBCL (n = 95) using RNeasy FFPE Kit (QIAGEN, 73504) according to the manufacturer’s instructions. For reverse transcription-quantitative polymerase chain reaction (RT-qPCR), the primers used for HAVCR2, LGALS9 and ACTIN were as follows: HAVCR2 forward, CAAAGGAGCCTGTCCTGTGT; HAVCR2 reverse, GCGGAAATCCCCATTTAGCC; LGALS9 forward, CTTTCATCACCACCATTCTG; LGALS9 reverse, ATGTGGAACCTCTGAGCACTG; ACTIN forward, CCGCGAGAAGATGACCCAGA; ACTIN reverse, GATAGCACAGCCTGGATAGCA. The samples were quantified using a Bio-Rad CFX manager and calculated using ACTIN as the reference gene.

### Statistical analyses

Statistical analyses were performed using the Statistical Package for the Social Sciences (SPSS) version 26.0 software (SPSS Corp., Chicago, IL, USA). Continuous and categorical data were analyzed using nonparametric and chi-squared tests, respectively. Spearman’s correlation coefficients were used to assess the correlation among gene and/or protein expression data. The cut-off values for HAVCR2/TIM3, LGALS9/Galectin-9, and clinical features were defined as the points where sensitivity and specificity were maximized in the receiver operating characteristic (ROC) curves for predicting overall survival (OS). Survival time was calculated using Kaplan–Meier analysis. A two-tailed p-value < 0.05 was considered statistically significant.

## Results

### The HAVCR2 expression and CD8^+^TILs exhaustion

**Fig. 1:**
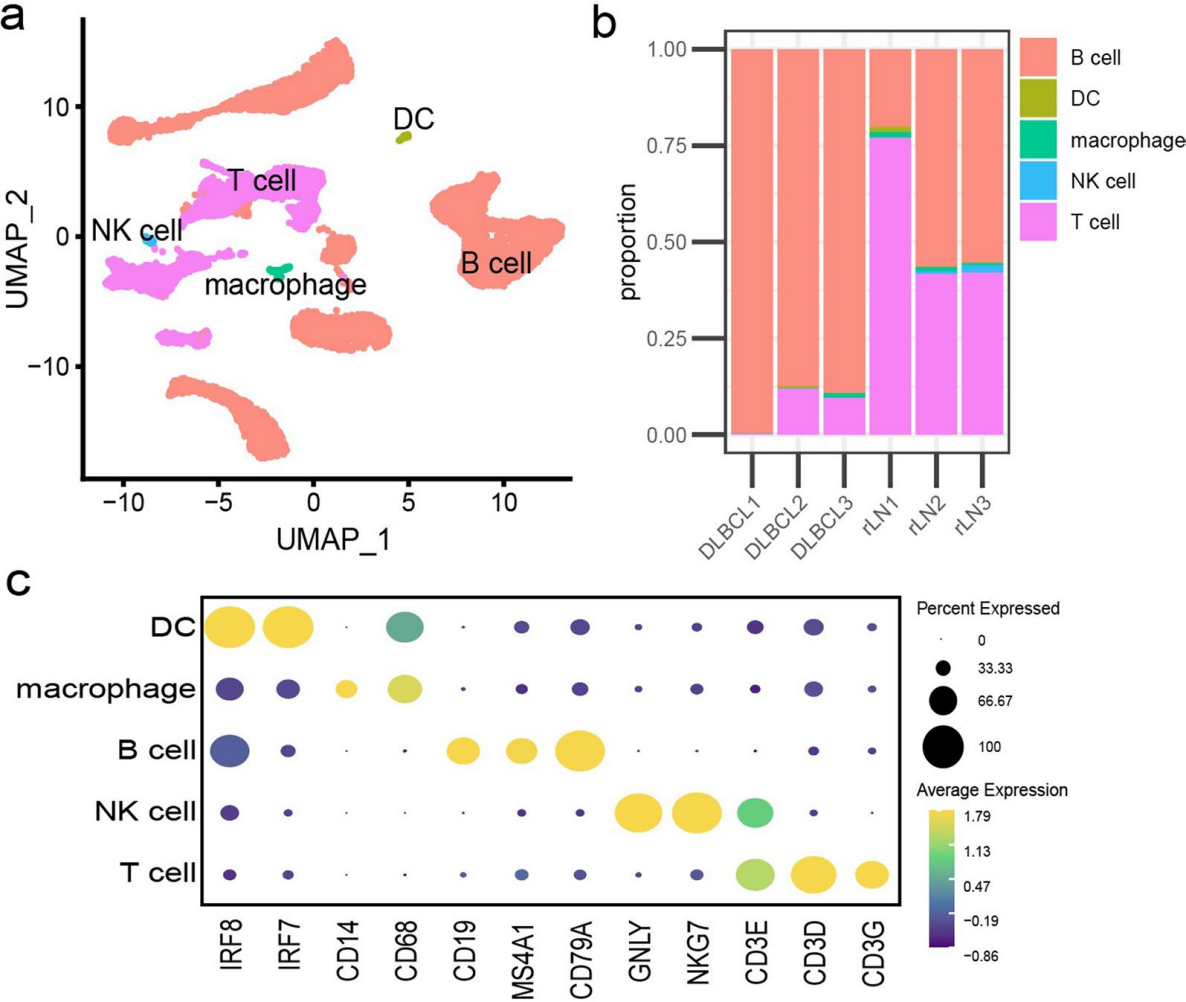
UMAP analysis (**a**), the proportion (**b**), and marker genes (**c**) for five major cell clusters in DLBCL (n = 3) and rLN samples (n = 3) detected using scRNA-seq. The major cell types included B cells (CD19, MS4A1, CD79A), T cells (CD3D, CD3E, CD3G), macrophages (CD14, CD68), DC (IRF7, IRF8) and NK cells (GNLY, NKG7)

**Fig. 2:**
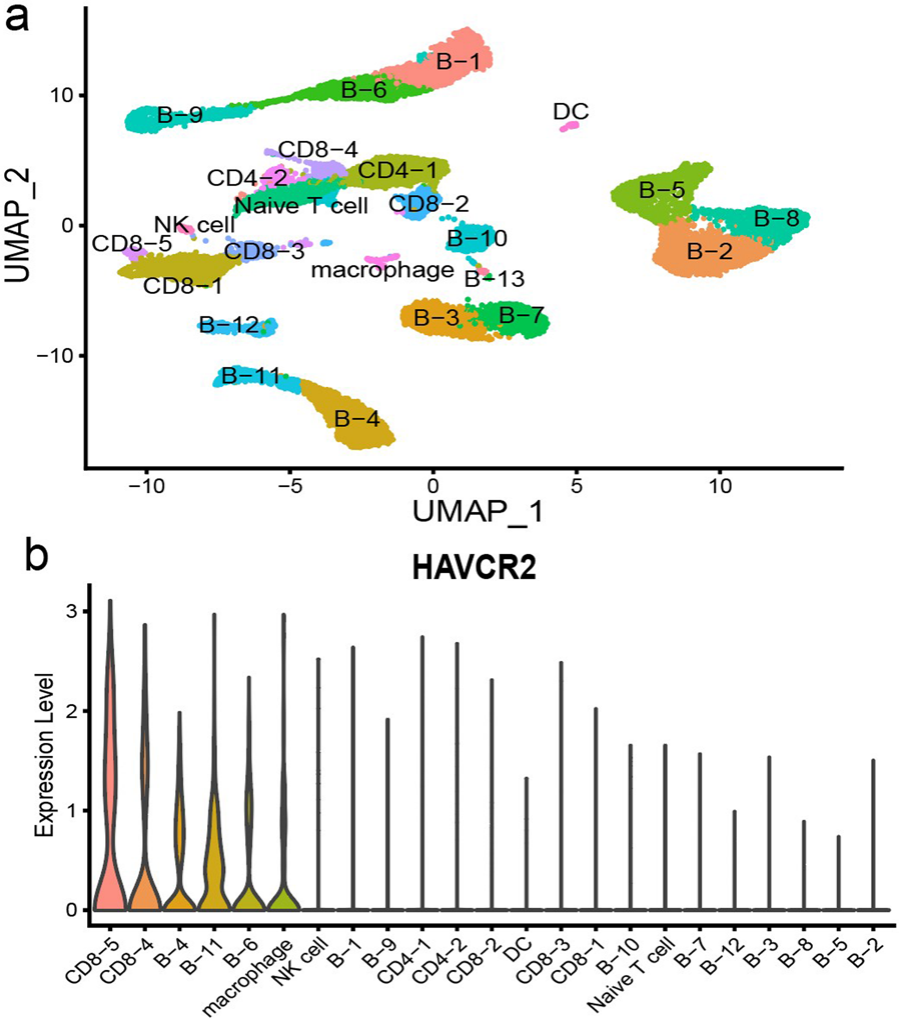
UMAP analysis (**a**) and the expression of HAVCR2 (**b**) for 24 cell clusters in DLBCL (n = 3) detected using scRNA-seq

After quality control and filtering, single-cell transcriptomes for 18,113 cells from the lymph node tissues of three DLBCL (DLBCL1, DLBCL2, DLBCL3) and three rLN (rLN1, rLN2, rLN3) were obtained for subsequent analysis. Through principal component analysis (PCA) and UMAP analysis, 5 major cell clusters (Fig. 1a) as well as the respective proportion in each sample were identified (Fig. 1b) using marker genes, including B cells (CD19, MS4A1, CD79A), T cells (CD3D, CD3E, CD3G), macrophages (CD14, CD68), DC (IRF7, IRF8) and NK cells (GNLY, NKG7) (Fig. 1c). The major cell clusters were further divided into 24 cell clusters, including naive T cells, CD4^+^TILs (n = 2, CD4-1, CD4-2), CD8^+^TILs (n = 5, CD8-1, CD8-2, CD8-3, CD8-4, CD8-5), B cells (n = 13, B-1, B-2, B-3, B-4, B-5, B-6, B-7, B-8, B-9, B-10, B-11, B-12, B-13), macrophage, DC, and NK cells (Fig. 2a). Among all clusters, HAVCR2 was mainly expressed in CD8-5 cells, followed by CD8-4 cells (Fig. 2b).

**Fig. 3:**
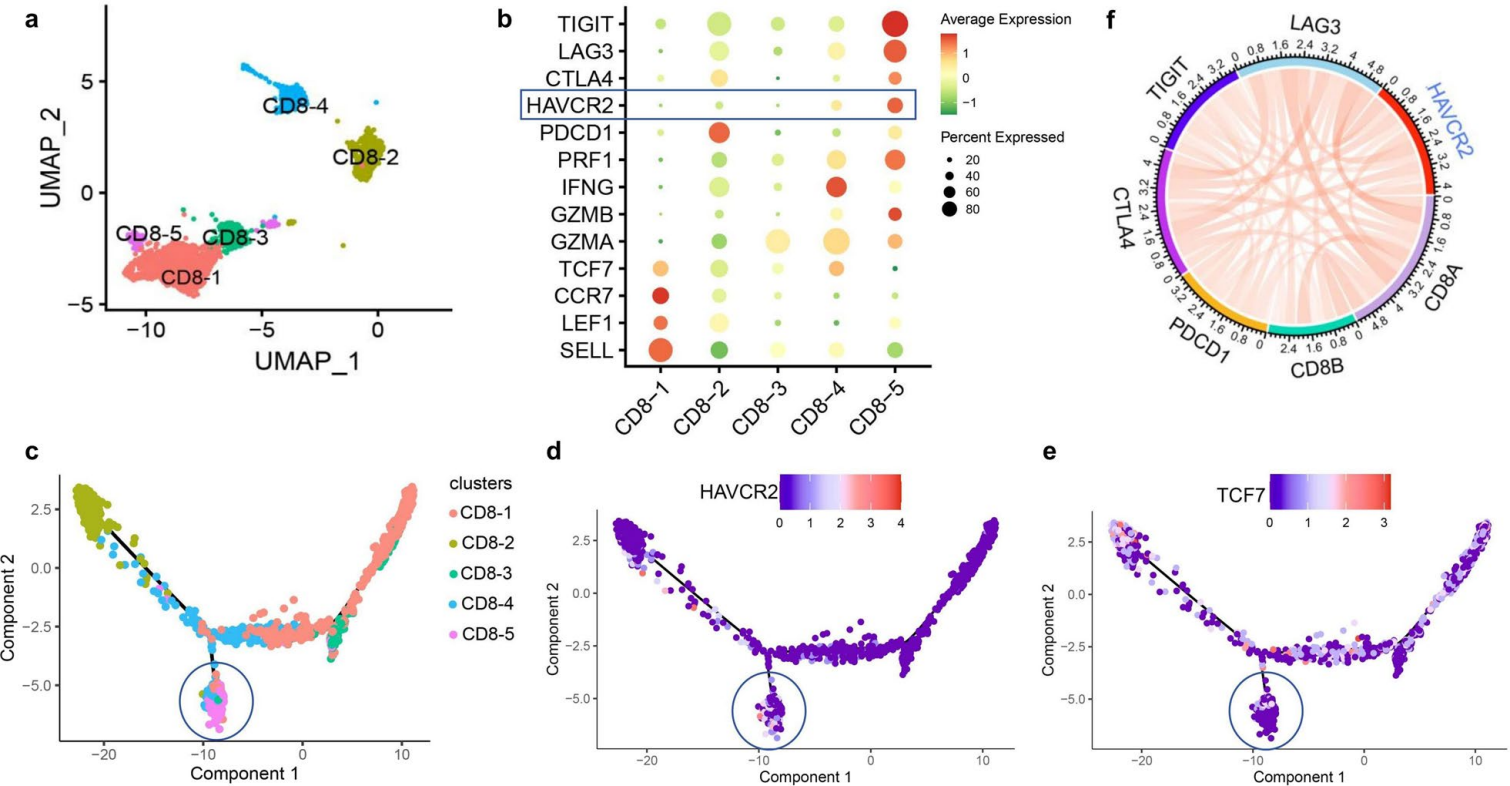
The HAVCR2 expression and CD8^+^TILs exhaustion in DLBCL. (**a**–**e** Single cell level). **a** UMAP analysis for different CD8^+^TILs clusters (CD8-1, CD8-2, CD8-3, CD8-4, CD8-5); **b** the expression of hallmark gene including differentiation, cytotoxicity and IC among different CD8^+^TILs clusters; **c**–**e** the cell trajectory analysis of CD8^+^TILs differentiation and the expression of exhaustion state-related characteristic gene (HAVCR2 and TCF7). Blue circle: mainly consists of CD8-5 cells (terminally exhausted state). **f** (Histology level) the correlation between HAVCR2 and CD8A, CD8B, and IC genes detected by RNA sequencing

Based on the expression of hallmark genes including differentiation, cytotoxic and IC, CD8-1, CD8-2, and CD8-3 cells were identified as naive, terminal effector, and early activated CD8^+^TILs, respectively (Fig. 3a, b). Multiple IC genes (CTLA4, HAVCR2, LAG3, TIGIT) were significantly over-expressed in CD8-5 cells, suggesting the exhaustion of CD8^+^TILs. Meanwhile, there were also certain expressions of IC genes in CD8-4 cells. Combined with the expression of TCF7 and HAVCR2, it is suggested that CD8-4 cells (TCF7^high^HAVCR2^low^) were progenitor exhausted CD8^+^TILs while CD8-5 cells (TCF7^low^HAVCR2^high^) were terminally exhausted CD8^+^TILs.

The results of developmental trajectories analysis of CD8^+^TILs showed that CD8-1 cells were at the early stage of differentiation, closely followed by CD8-3 cells. Then there were two differentiation trajectories, in which trajectory 1 ended with CD8-2 cells, while trajectory 2 ended with CD8-5 cells accompanied with low expression of TCF7 and high expression of HAVCR2, and CD8-4 cells distributed along with both trajectories (Fig. 3c–e).

The results of correlation analysis based on bulk RNA sequencing data of DLBCL showed that HAVCR2 was positively correlated with CD8A (r = 0.46, p < 0.001), CD8B (r = 0.28, p < 0.001), LAG3 (r = 0.56, p < 0.001), PDCD1 (r = 0.33, p < 0.001), CTLA4 (r = 0.44, p < 0.001) and TIGIT (r = 0.35, p < 0.001) (Fig. 3f).

### The key ligand of TIM3-mediated CD8^+^TILs exhaustion

**Fig. 4:**
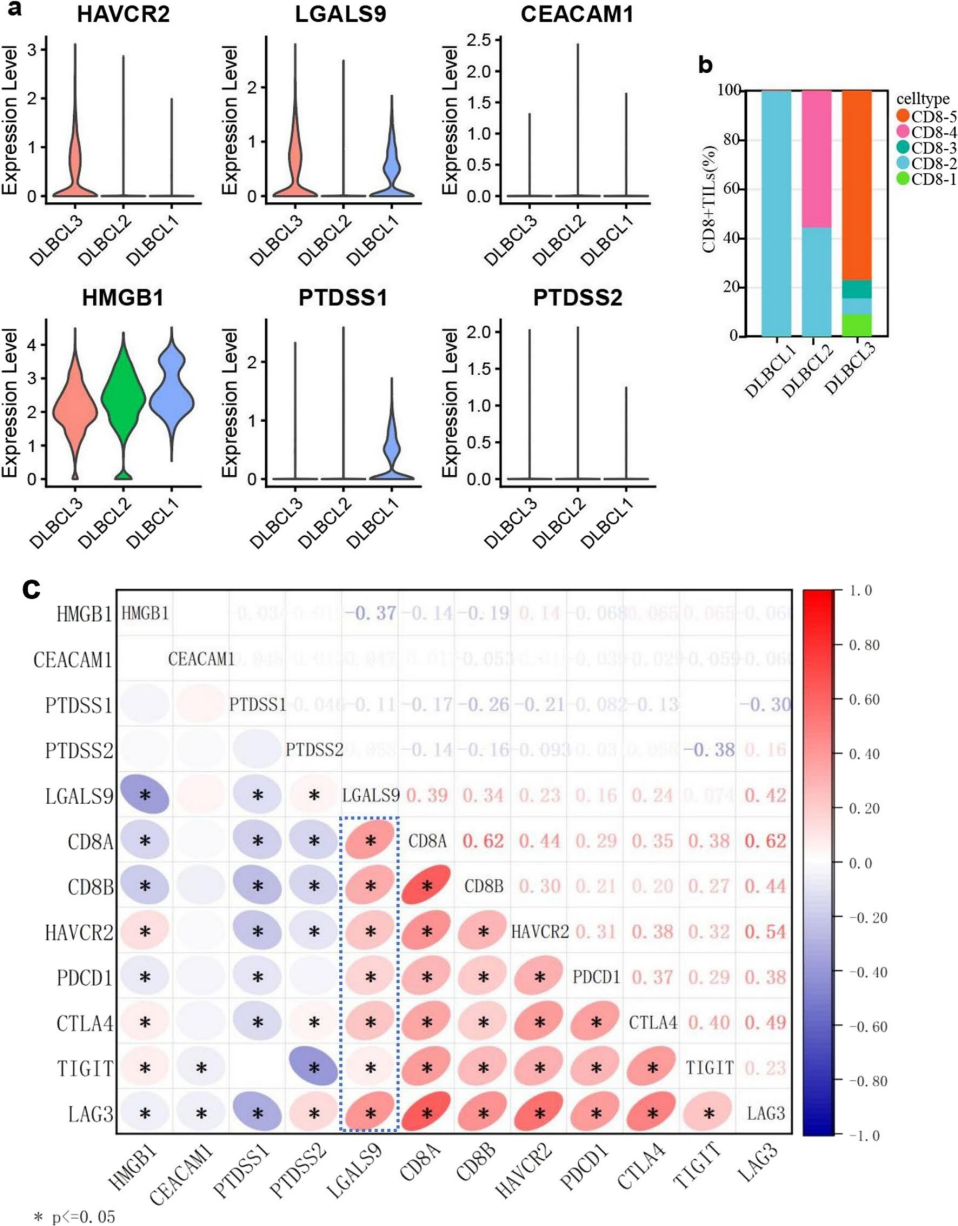
The key ligand of TIM3-mediated CD8^+^TILs exhaustion in DLBCL. The correlation among TIM3 and its ligand genes (LGALS9, HMGB1, CEACAM1, PTDSS1, PTDSS2) (**a**), and the composition of CD8^+^TILs detected by scRNA-seq. Red: CD8-5 cells (terminal exhausted CD8^+^TILs) (**b**); **c** correlation between the ligand of TIM3 and CD8A, CD8B, and IC gene expression detected by RNA sequencing

The correlation between the expression of TIM3-ligand genes (LGALS9, HMGB1, CEACAM1, PTDSS1, PTDSS2) and the proportion of exhausted CD8^+^TILs in DLBCL was analyzed by scRNA-seq (Fig. 4a, b). The results showed that the expression of LGALS9 and HAVCR2 were both the highest in DLBCL3, in which the main composition of CD8^+^TILs were CD8-5 cells (terminally exhausted state). Few expressions of HAVCR2 and a low proportion of CD8-5 cells were detected in DLBCL1 and DLBCL2 with high expression of HMGB1 or PTDSS1. In addition, the expression of CEACAM1 and PTDSS2 were both low in all cases.

Further correlation analysis among the gene expression of TIM3-ligands, CD8^+^TILs marker (CD8A, CD8B), and IC was performed utilizing the bulk RNA sequencing data of DLBCL. The results showed that only LGALS9 was positively correlated with the expression of all those genes related to CD8^+^TILs exhaustion (Fig. 4c).

### The potential pathway of TIM3-mediated CD8^+^TILs exhaustion

**Fig. 5:**
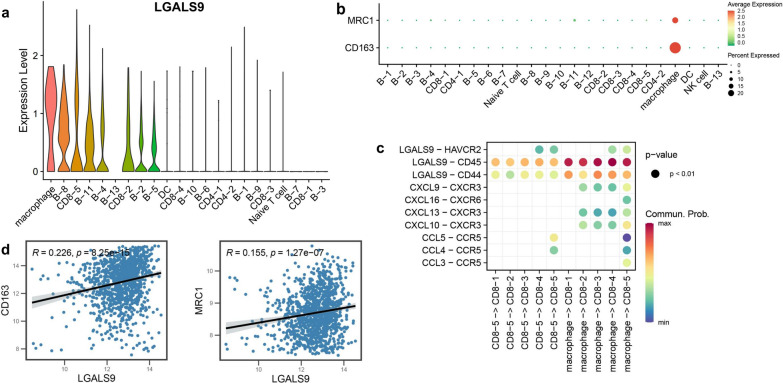
The potential pathway of TIM3-mediated CD8^+^TILs exhaustion in DLBCL. (**a**–**c** Single cell level). The expression of LGALS9 (**a**) and the characteristic genes of M2 macrophage (CD163 and MRC1/CD206) (**b**) for 24 cell clusters in DLBCL; **c** interaction network of TIM3/Galectin-9 pathway in DLBCL; **d** (histology level) the correlation between LGALS9 and CD163, and MRC1/CD206 detected by RNA sequencing

The results of scRNA-seq showed that among all the cell clusters in DLBCL, the expression of LGALS9 was the highest in macrophages, followed by B-8, CD8-5, B-11, and B-4 cells (Fig. 5a). The over-expression of CD163 and CD206/MRC1 was detected in macrophages from DLBCL (Fig. 5b). Cellular communication analysis by CellChat demonstrated that there were many interactions among macrophages and five clusters of CD8^+^TILs in DLBCL (Fig. 5c), including a variety of ligand-receptor pairs related to chemokine, such as CXCL9-CXCR3, and macrophages interacted with CD8-5 cells and CD8-4 cells through HAVCR2-LGALS9. Additionally, the HAVCR2-LGALS9 interactions between CD8-5 cells and CD8-5 cells and/or CD8-4 cells were also found. The results of correlation analysis based on bulk RNA sequencing data of DLBCL showed that LGALS9 was positively correlated with CD163 (r = 0.226, p < 0.01) and CD206/MRC1 (r = 0.155, p < 0.01) (Fig. 5d).

### The protein and gene expression of TIM3/Galectin-9 pathway in DLBCL

**Fig. 6:**
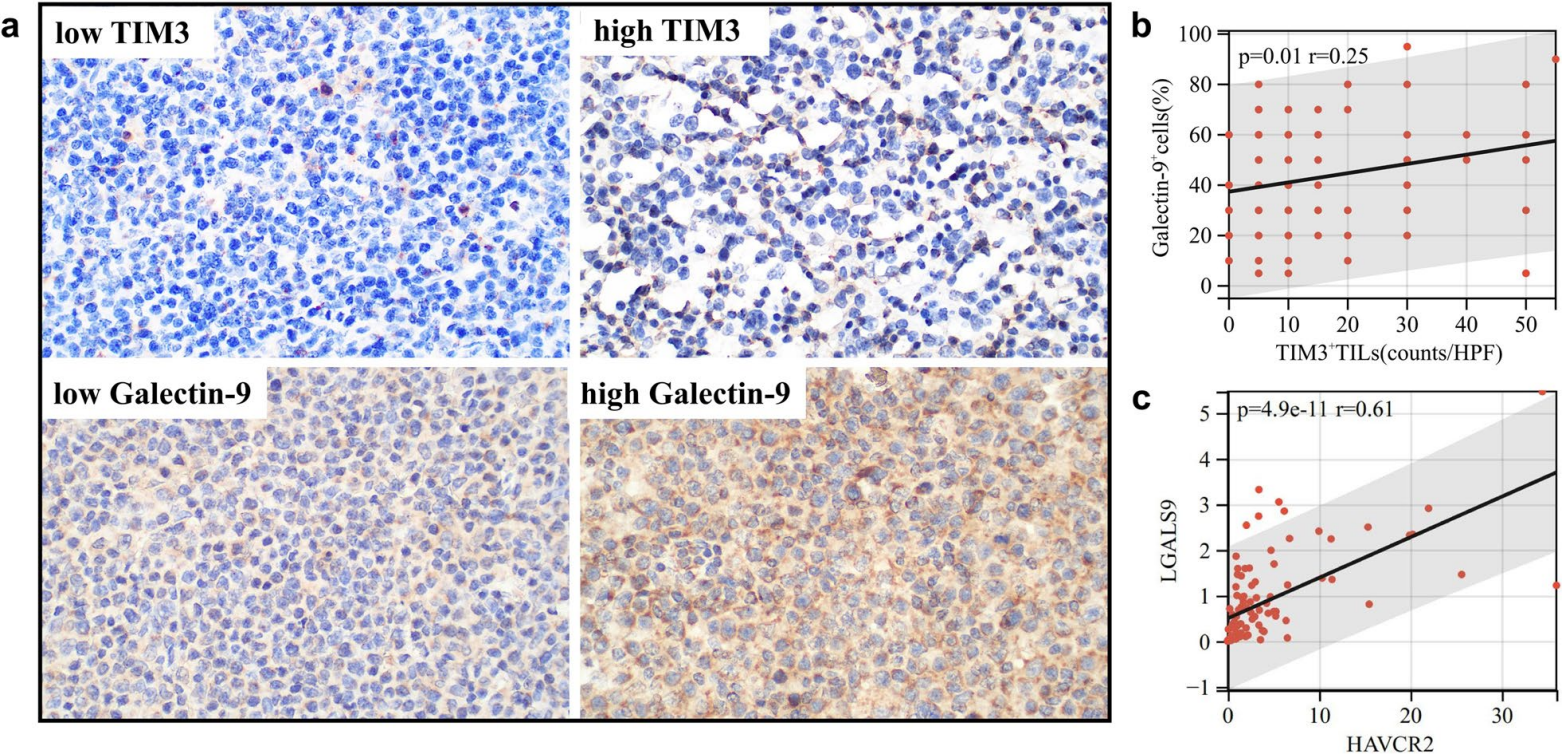
The protein and gene expression of TIM3/Galectin-9 pathway in DLBCL. **a** The high and low expression of TIM3 and Galectin-9 detected by IHC; **b** the correlation between the protein expression of TIM3 and Galectin-9 detected by IHC (n = 100); **c** the correlation between the gene expression of HAVCR2 and LGALS9 detected by RT-qPCR (n = 95)

The basic information of DLBCL patients (n = 100) is summarized in Table 1. The median counts of TIM3^+^ TILs were 10/HPF (0–55/HPF), and the median percent of Galectin-9^+^ cells was 40% (5–95%) detected by IHC (Fig. 6a). The protein expression of TIM3 was positively correlated with Galectin-9 in DLBCL (r = 0.25, p = 0.01) (Fig. 6b). The gene expression of HAVCR2 also showed a positive correlation with LGALS9 in DLBCL detected by RT-qPCR (r = 0.61, p < 0.01) (Fig. 6c).

**Table 1:** Baseline characteristics of patients with DLBCL

Characteristic	No	In group (%)
Age, mean (range)	61.5 (25–88)	
Age		
> 60 years	55/100	55
≤ 60 years	45/100	45
Gender		
Male	57/100	57
Female	43/100	43
COO		
GCB	31/100	31
Non-GCB	69/100	69
PS		
0–1	70/95	73.7
2–5	25/95	26.3
Stage		
I/II	38/97	39.2
III/IV	59/97	60.8
IPI		
0–1	45/96	46.9
2–5	51/96	53.1
B-symptom		
Yes	32/98	32.7
No	66/98	67.3
Primary site		
Nodal	65/97	67
Extranodal	32/97	33
LDH > 220 IU/L		
Yes	39/68	57.4
No	29/68	42.6
Treatment		
CHOP-based therapy	77/90	85.6
Other therapy	13/90	14.4
Response to CHOP		
CR + PR	40/61	65.6
SD + PD	21/61	34.4

*COO* cell of origin, *GCB* germinal center B-cell-like, *PS* performance status, *LDH* lactate dehydrogenase, *CHOP* cyclophosphamide, doxorubicin, vincristine, prednisone, *CR* complete remission, *PR* partial remission, *SD* stable disease, *PD* progressive disease

### The correlation between TIM3/Galectin-9 pathway and clinical features

According to TIM3/Galectin-9 enrichment, patients were divided into two groups by the median protein expression of TIM3 and Galectin-9: high enrichment group for patients with TIM3^+^ TILs > 10/HPF and Galectin-9^+^ cells > 40% and low enrichment group for the remaining patients. The correlation between the two groups and clinical features is shown in Additional file 1: Table S1. Compared with the low enrichment group, patients in the high enrichment group were more likely to present with advanced-stage (III/IV) (87% vs. 52.7%, p = 0.003), and have higher PS score (2–5) (55.5% vs. 20.5%, p = 0.028), and lower response rate to CHOP (complete response and partial response) (41.7% vs. 76.1%, p = 0.035).

### The significance of TIM3/Galectin-9 pathway in prognosis evaluation

**Fig. 7:**
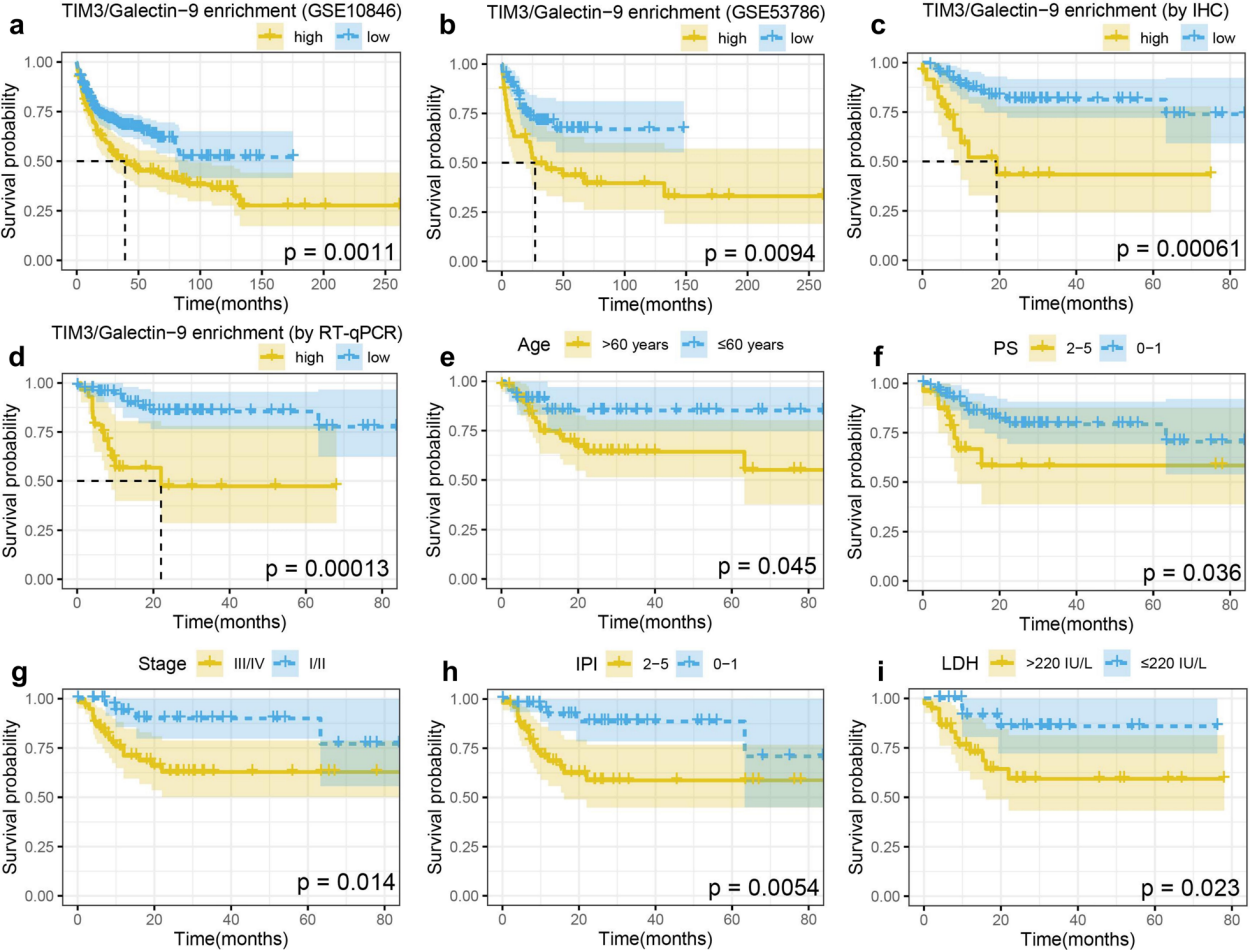
The survival analysis in DLBCL. **a**, **b** Kaplan–Meier estimates of OS rate for TIM3/Galectin-9 enrichment by RNA-sequencing in GEO data (GSE10846, n = 412; GSE53786, n = 119); **c**–**h** Kaplan–Meier estimates of OS rate for TIM3/Galectin-9 enrichment by IHC (**c**) and RT-qPCR (**d**), age (**e**), PS (**f**), stage (**g**), IPI (**h**) and LDH (**i**) in our cohort

The impact of TIM3/Galectin-9 enrichment on the prognosis of DLBCL patients was analyzed based on the training cohort of GSE10846 (n = 412) and GSE53786 (n = 119) and validated by our cohort (n = 100). The survival analysis of the training cohort suggested that patients with high TIM3/Galectin-9 enrichment demonstrated shorter OS compared to those with low TIM3/Galectin-9 enrichment (Fig. 7a, b). In our cohort, the result of univariate analysis showed that high TIM3/Galectin-9 enrichment both on protein (p < 0.001) (Fig. 7c) and transcription level (p < 0.001) (Fig. 7d), age > 60 years (p = 0.045) (Fig. 7e), PS 2–5 (p = 0.036) (Fig. 7f), stage III/IV (p = 0.014) (Fig. 7g), IPI 2–5 (p = 0.005) (Fig. 7h), and LDH > 220 IU/L (p = 0.023) (Fig. 7i) were significant risk factors of OS, and the result of multivariate analysis revealed that high TIM3/Galectin-9 enrichment (p = 0.018) and LDH > 220 IU/L (p = 0.044) were independent prognostic factors for DLBCL patients (Table 2).

**Table 2:** Univariate and multivariate Cox regression analyses with relative risk of OS estimated as hazard ratios with 95% confidence intervals and P values by putative prognostic factors in de dovo DLBCL patients

	Univariate		Multivariate	
	HR (95%)	*p*	HR (95%)	*p*
TIM3/Galectin-9 enrichment				
High vs low	0.257:0.111–0.593	**0.001**	0.275:0.071–1.066	**0.018**
Age				
> 60 vs ≤ 60	0.4:0.157–1.014	**0.045**	0.372:0.094–1.478	0.16
Gender				
Male vs female	0.884:0.383–2.043	0.773	–	–
COO				
Non-GCB vs GCB	0.322:0.096–1.084	0.053	–	–
PS				
2–5 vs 0–1	0.397:0.163–0.969	**0.036**	0.351:0.079–1.556	0.168
Stage				
III/IV vs I/II	0.282:0.095–0.834	**0.014**	0.25:0.032–1.972	0.188
IPI				
2–5 vs 0–1	0.267:0.098–0.726	**0.005**	6.974:0.501–97.122	0.148
B symptoms				
Yes vs no	0.592:0.251–1.395	0.793	–	–
Primary site				
Nodal vs extranodal	0.917:0.389–2.164	0.843	–	–
LDH > 220 IU/L				
Yes vs no	0.256:0.072–0.910	**0.023**	0.146:0.018–1.185	**0.044**

Statistical significance (*p* < 0.05) is indicated by boldface font

### The correlation between TIM3/Galectin-9 pathway and TME composition

**Fig. 8:**
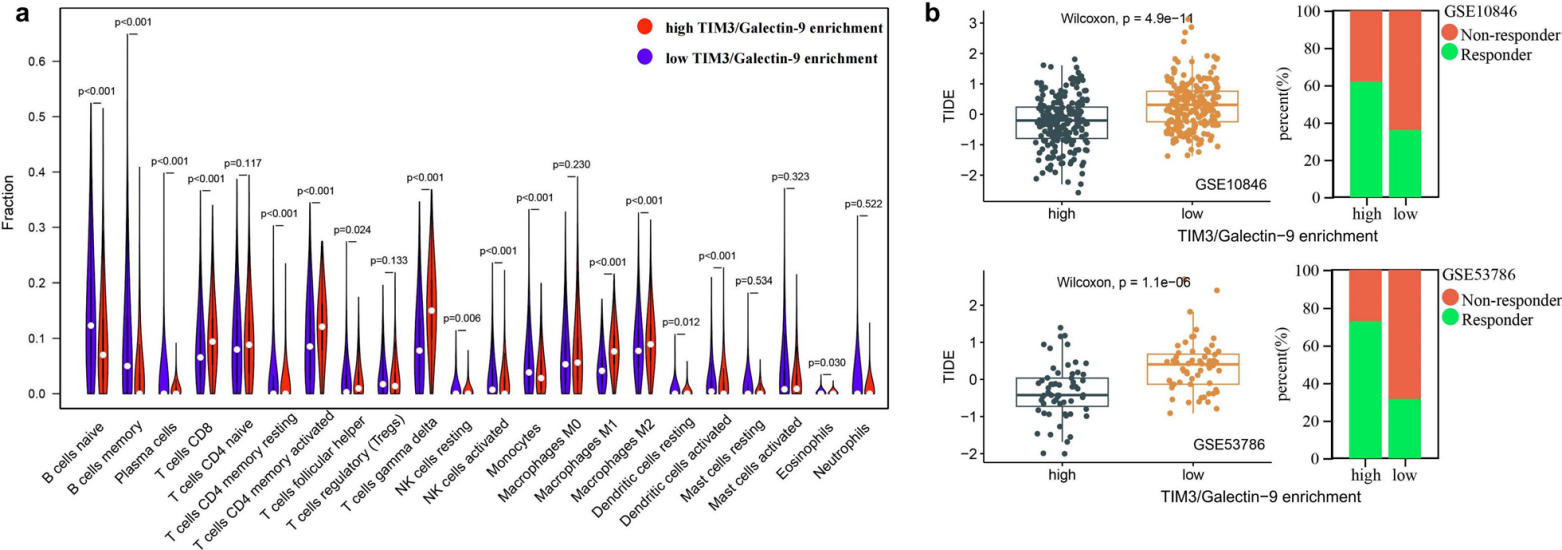
The significance of TIM3/Galectin-9 pathway in DLBCL evaluated by RNA sequencing. **a** The correlation between TIM3/Galectin-9 enrichment and TME composition detected by CIBERSORT; **b** the correlation between TIM3/Galectin-9 enrichment and TIDE scores (left). The predicted response to immune checkpoint blockade therapy in high and low TIM3/Galectin-9 enrichment groups (right)

The correlation between TIM3/Galectin-9 enrichment and TME composition analyzed by CIBERSORT is shown in Fig. 8a. Compared with the low TIM3/Galectin-9 enrichment group, the high TIM3/Galectin-9 enrichment group had higher proportions of CD8 T cells and M2 macrophages, and lower proportions of naive B cells, memory B cells, plasma cells, and NK cells.

### The correlation between TIM3/Galectin-9 pathway and TIDE scores

The correlation between TIM3/Galectin-9 enrichment and ICB response analyzed by TIDE is shown in Fig. 8b. Compared with the low TIM3/Galectin-9 enrichment group, the TIDE scores were lower in the high TIM3/Galectin-9 enrichment group, accompanied with a higher predicted response rate of ICB therapy.

### The possible regulatory mechanism of TIM3/Galectin-9 pathway

**Fig. 9:**
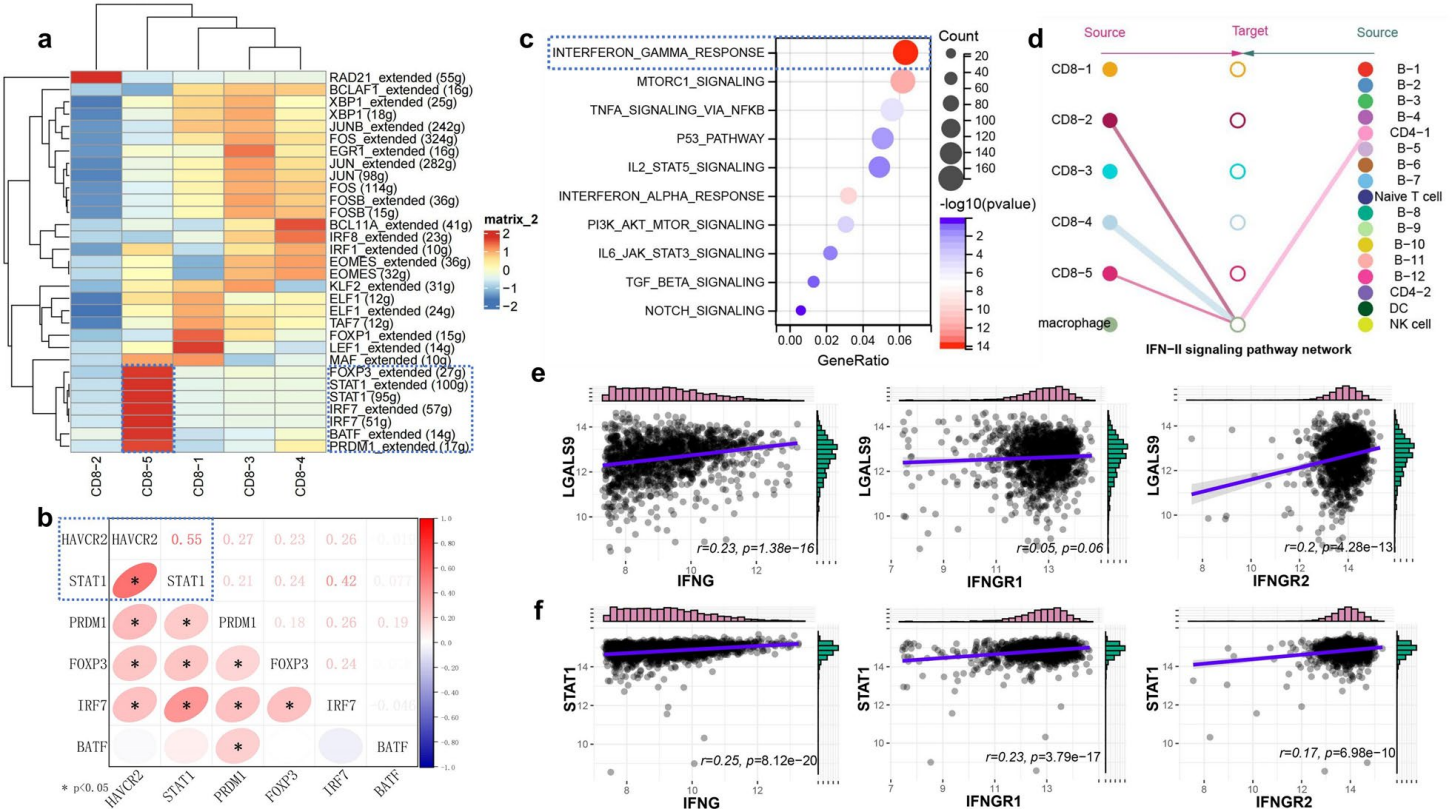
The possible regulatory mechanism of TIM3/Galectin-9 pathway in DLBCL. (**a**, **c**, **d** Single cell level, **b**, **e**, **f** histology level). **a** The TFs activated in different cell clusters of CD8^+^TILs predicted by SCENIC; **b** the correlation between HAVCR2 and TFs activated in CD8-5 cells detected by RNA sequencing; **c** functional enrichment analysis of LGASL9-coexpressed genes in macrophage by Hallmark gene set; **d** interaction network of IFNG-IFNGR1/IFNGR2 pathway among macrophages and other clusters in DLBCL; **e** the correlation between LGALS9 and IFNG, IFNGR1, and IFNGR2 detected by RNA sequencing; **f** the correlation between STAT1 and IFNG, IFNGR1, and IFNGR2 detected by RNA sequencing

SCENIC analysis using scRNA-seq data predicted 5 main TF motifs, including FOXP3, STAT1, IRF7, BATF, and PRDM1, to be activated in CD8-5 cells (enriched in HAVCR2) compared with all the other cell clusters in CD8^+^TILs (Fig. 9a). Further analysis using bulk RNA sequencing data of DLBCL identified that STAT1 has the strongest positive correlation with HAVCR2 expression (r = 0.55, p < 0.01) (Fig. 9b).

The correlation between LGALS9 expression and all the other genes in macrophages from DLBCL was analyzed by scRNA-seq data. The results of Hallmark enrichment analysis of the genes (n = 500) with the strongest positive correlation with LGALS9 (Additional file 2: Table S2) showed that multiple pathways were enriched such as IFN-γ response, MTORC1 signaling, and TNF-α signaling via NFKB pathways, among which IFN-γ response pathway was significantly activated (Fig. 9c). Further cellular communication analysis showed that CD8^+^TILs (including CD8-2, CD8-4, and CD8-5 cells) and CD4^+^TILs (CD4-1 cells) could interact with macrophages through IFNG-IFNGR1/IFNGR2 pathway (Fig. 9d). The results of correlation analysis based on bulk RNA sequencing data of DLBCL showed that LGALS9 was positively correlated with IFNG (r = 0.23, p < 0.01) and IFNGR2 (r = 0.2, p < 0.01) instead of IFNGR1 (r = 0.05, p = 0.06) (Fig. 9e). In addition, STAT1 showed a positive correlation with IFNG (r = 0.25, p < 0.01), IFNGR1 (r = 0.23, p < 0.01), and IFNGR2 (r = 0.17, p < 0.01) (Fig. 9f).

## Discussion

DLBCL is the most common subtype of aggressive lymphoma with certain patients who cannot benefit from the current treatment [2], and novel therapeutic targets were needed to explore. The disorder of TME plays a vital role in the development and metastasis of DLBCL [5, 6], among which CD8^+^TILs are indispensable for tumor eradication and prognosis evaluation [7, 8]. Recently, Zhao et al. demonstrated the exhaustion status of CD8^+^TILs in DLBCL and constructed a prognostic model based on the associated genes [23]. Meanwhile, previous studies have shown that the over-expression of TIM3 is related to CD8^+^TILs exhaustion in DLBCL [13, 21]. As a novel IC, TIM3 can induce exhaustion and inhibit the function of CD8^+^TILs in ways different from those of PD-1 and CTLA-4 [24], providing a valuable strategy for patients resistant to current treatment including ICB therapy. However, the mechanism of TIM3-mediated CD8^+^TILs exhaustion in DLBCL remains to be explored, and further investigation may be helpful for improving the prognosis of DLBCL patients.

Our study demonstrated that CD8^+^TILs, especially the terminally exhausted state characterized by low expression of TCF7 [25], were the main cluster that expressed TIM3, suggesting the central role of TIM3 in the impairment of anti-tumor function, and may induce the immune escape of DLBCL. Further analysis suggested that Galectin-9 is the key ligand of TIM3-mediated CD8^+^TILs exhaustion in DLBCL. In many solid tumors and lymphohematopoietic neoplasms, TIM3/Galectin-9 pathway is closely related to CD8^+^TILs exhaustion, tumor immune evasion, and poor prognosis, such as breast cancer [26], glioma [27], and acute myeloid leukemia [28]. Besides, our study confirmed the positive correlation between Galectin-9 and multiple ICs of CD8^+^TILs in DLBCL. It is worth noting that Galectin-9 could also interact with other ICs expressed by CD8^+^TILs, such as PD-1 and VISTA, leading to the progressive deterioration of effector function of CD8^+^TILs [29, 30]. Combined with the literature and the results of our study, the potential role of TIM3/Galectin-9 pathway in the CD8^+^TILs exhaustion was implied.

To further investigate the significance of TIM3/Galectin-9 pathway in DLBCL, we performed immune cell infiltration and clinicopathological analysis. The results showed that patients with high TIM3/Galectin-9 enrichment indicated the disturbance of TME, including the increase of M2 macrophages, and decrease of naive B cells, memory B cells, plasma cells, and NK cells, implying the immunosuppression of TME [31] and the impairment of immune function [32, 33]. This may be the important cause for the inferior prognosis and severe clinical manifestations such as high PS score, and advanced stage in DLBCL patients with high TIM3/Galectin-9 enrichment. Taken together, these results further emphasized the significance of CD8^+^TILs exhaustion induced by TIM3/Galectin-9 pathway in the immune deficiency and tumor escape of DLBCL.

Meanwhile, our study demonstrated that Galectin-9 was mainly expressed in M2 macrophage in DLBCL, which could interact with CD8^+^TILs through TIM3/Galectin-9 pathway. Besides, macrophages could recruit CD8^+^TILs to the TME through a variety of ligand-receptor pair interactions related to chemokine, such as CXCL9-CXCR3. M2 macrophages are typical immunosuppressive cells and can promote the progression of tumor through multiple mechanisms, such as secreting suppressive cytokines, interfering with humoral and cellular immune function [31], and increasing the infiltration of Treg [34]. It has been reported that M2 macrophages highly expressed Galectin-9 [35, 36], and Galectin-9 could also polarize macrophages toward the M2 phenotype, further inhibiting the anti-tumor function of T lymphocytes through TIM3/Galectin-9 pathway [27, 37, 38]. Therefore, we hypothesized that M2 macrophages in DLBCL not only can increase the infiltration of CD8^+^TILs but also may induce CD8^+^TILs exhaustion through TIM3/Galectin-9 pathway. Interestingly, our study found that there was certain expression of Galectin-9 in the terminally exhausted CD8^+^TILs in DLBCL, which might aggravate the exhaustion of CD8^+^TILs through TIM3/Galectin-9 pathway and further weaken the anti-tumor function of CD8^+^TILs. In addition, the expression of Galectin-9 was also detected in multiple types of B cells, which may include normal and malignant cells, revealing the complex network between tumor cells and background cells, and further suggesting the significance of TIM3/Galectin-9 pathway in DLBCL.

To further explore the regulatory mechanism of TIM3/Galectin-9 pathway in DLBCL, the significantly activated TFs in exhausted CD8^+^TILs, and Galectin-9-coexpressed genes in macrophages were analyzed on single cell and validated on histology level. Our results showed that TFs including STAT1, PRDM1, and IRF7 were activated in exhausted CD8^+^TILs in DLBCL. These TFs are closely associated with TIM3 expression [39, 40] and CD8^+^TILs exhaustion [41, 42], among which STAT1 was identified as the critical TF that regulated the expression of TIM3 in CD8^+^TILs from DLBCL.

In macrophages, the IFN-γ response, MTORC1 signaling, and TNF-α signaling via NFKB pathways were enriched. Among these pathways, IFN-γ response pathway that involved the interaction among macrophages, CD8^+^TILs, and CD4^+^TILs, was significantly activated. These results are consistent with the previous study that IFN-γ could induce the production of Galectin-9 in macrophages [29, 43]. Moreover, the positive correlation between STAT1 and IFN-γ response pathway was also confirmed in DLBCL. Despite the positive role in the immune response in many tumors, IFN-γ can also induce the expression of STAT1 [42] and the exhaustion of T lymphocytes demonstrated by the over-expression of TIM3 [44]. Therefore, we speculate that IFN-γ signaling pathway regulated the expression of TIM3/Galectin-9 in DLBCL which deserves further investigation. In addition, the metabolic status, such as glycolysis, of exhausted CD8^+^TILs is reported to be abnormally active and is related to the resistance to ICB therapy [45]. A recent study has shown that the abnormal expression of glycolysis-related signature genes in DLBCL is related to the infiltration of CD8^+^TILs and TIM3 expression [46], suggesting the possible role of glycolysis in TIM3/Galectin-9 pathway mediated CD8^+^TILs exhaustion in DLBCL.

The blockade of TIM3/Galectin-9 pathway, to potentiate the anti-tumor function of CD8^+^TILs and eradicate malignant cells has already revolutionized the treatment of many solid tumors [47, 48] while rare in DLBCL. Our study showed that patients with high TIM3/Galectin-9 enrichment suggested poor response to CHOP-based chemotherapy. This may be caused by the impaired immune response of exhausted CD8^+^TILs induced by high TIM3/Galectin-9 enrichment, thus leading to the formation of the immunosuppressive tumor microenvironment, and the immune escape and proliferation of tumor cells. Meanwhile, patients with high TIM3/Galectin-9 enrichment may be susceptible to ICB treatment. In addition, previous studies have pointed out that anti-Galectin-9 therapy may have a stronger therapeutic effect compared with mono-therapy targeted on IC due to its interaction with multiple ICs [29, 30], and may help to prolong the survival of patients with resistance to PD-1/PD-L1 blockade therapy [15]. In brief, TIM3/Galectin-9 enrichment could serve as a valuable indicator for the response to ICB therapy and a novel promising therapeutic target in DLBCL.

**Fig. 10:**
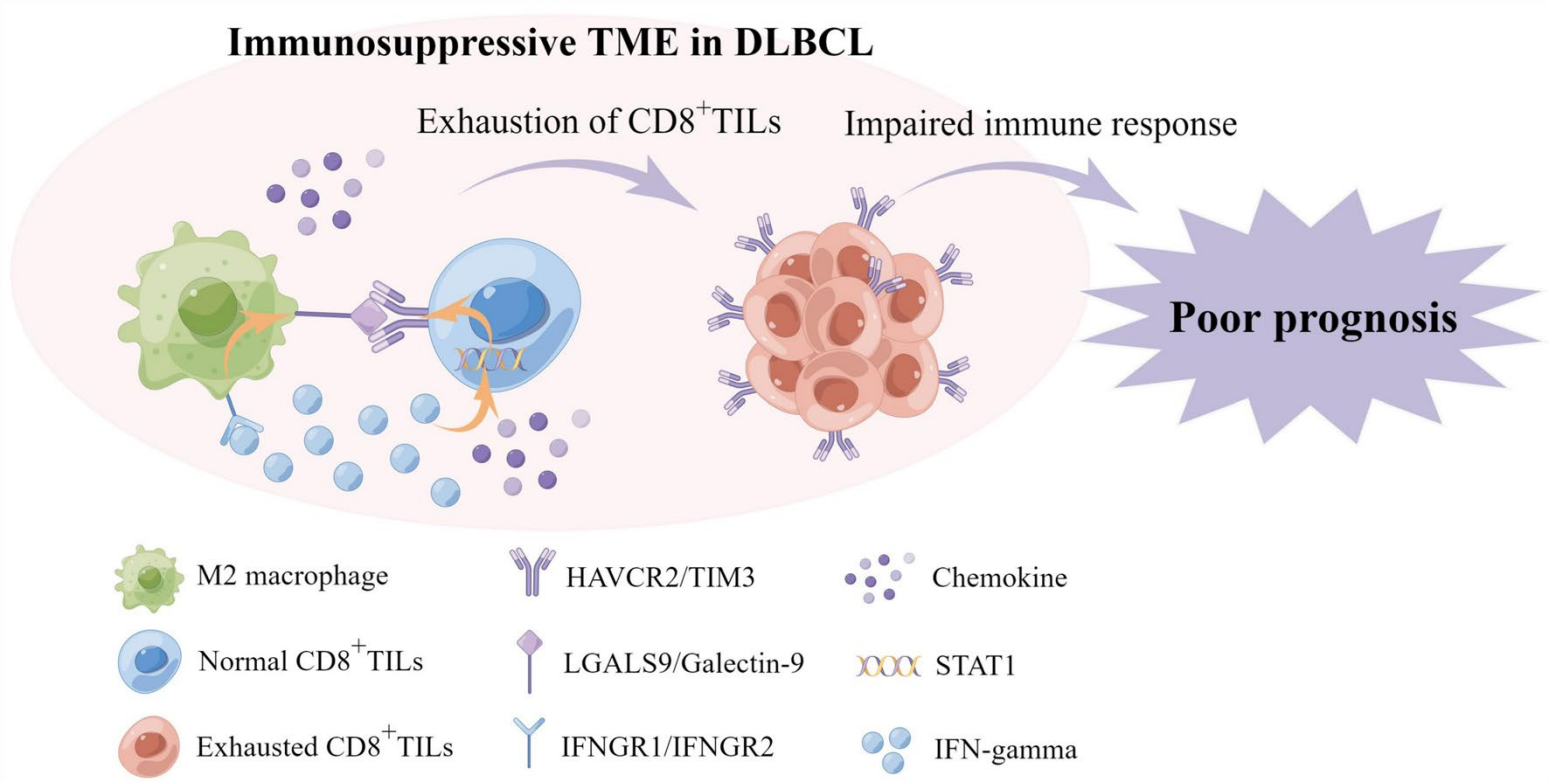
The predicted model of CD8^+^TILs exhaustion mediated by M2 macrophage through TIM3/Galectin-9 pathway and induced poor prognosis in DLBCL

In conclusion, our study demonstrates that Galectin-9, mainly expressed in M2 macrophages, is the key ligand of TIM3 and can induce the exhaustion of CD8^+^TILs through TIM3/Galectin-9 pathway. High TIM3/Galectin-9 enrichment is related to immunosuppressive TME, inferior prognosis, and severe clinical manifestations, which may be caused by the impairment of the anti-tumor function of exhausted CD8^+^TILs. High TIM3/Galectin-9 enrichment also suggested the poor response to treatment and could predict the clinical efficacy of ICB therapy in DLBCL. Furthermore, the TIM3/Galectin-9 enrichment in DLBCL may be regulated by IFN-γ signaling pathway (Fig. 10). Overall, our study highlights that TIM3/Galectin-9 pathway plays an essential role in CD8^+^TILs exhaustion and the immune escape of DLBCL, and is a valuable prognosticator and can facilitate the development of novel immunotherapy. Meanwhile, further functional studies and validations in larger cohorts are required to confirm these findings.

### Supplementary Information

**Additional file 1: Table S1.** The correlation between TIM3/Galectin-9 enrichment and clinical features in DLBCL.

**Additional file 2: Table S2.** The co-expressed genes (n = 500) with LGALS9 in macrophages in DLBCL detected by scRNA-seq.

## Data Availability

The datasets presented in this study can be found in online repositories. The names of the repository/repositories and accession number(s) can be found below: RNA sequencing data: GEO database, https://www.ncbi.nlm.nih.gov/geo/, GSE181063, GSE10846 and GSE53786. Single-cell RNA sequencing data: heiDATA database, https://heidata.uni-heidelberg.de/dataset.xhtml?persistentId=doi:10.11588/data/VRJUNV.
