# Supplementary material for: Diffuse large B-cell lymphoma: the significance of CD8+ tumor-infiltrating lymphocytes exhaustion mediated by TIM3/Galectin-9 pathway
Source: J Transl Med. 2024 Feb 18;22:174. doi: 10.1186/s12967-024-05002-3 (PMC10874540; doi:10.1186/s12967-024-05002-3)
Supplement: Supplementary file 1 — Additional file 1: Table S1. The correlation between TIM3/Galectin-9 enrichment and clinical features in DLBCL. [file 12967_2024_5002_MOESM1_ESM.docx]

Table S1. The correlation between TIM3/Galectin-9 enrichment and clinical features in DLBCL.

| Characteristic | low TIM3/Galectin-9 enrichment | high TIM3/Galectin-9 enrichment | *p* |
| --- | --- | --- | --- |
| N | 76 | 24 |  |
| Age, mean (range) | 59.8(25-88) | 67.1(30-85) |  |
| Age |  |  | 0.139 |
| >60 years | 39/76(51.3%) | 16/24(66.7%) |  |
| ≤60 years | 37/76(48.7%) | 8/24(33.3%) |  |
| Gender |  |  | 0.1 |
| Male | 47/76(61.8%) | 10/24(41.7%) |  |
| Female | 29/76(38.2%) | 14/24(58.3%) |  |
| COO |  |  | 0.519 |
| GCB | 24/76(31.6%) | 7/24(29.2%) |  |
| Non-GCB | 52/76(68.4%) | 17/24(70.8%) |  |
| PS |  |  | **0.028** |
| 0-1 | 58/73(79.5%) | 12/22(54.5%) |  |
| 2-5 | 15/73(20.5%) | 10/22(45.5%) |  |
| Stage |  |  | **0.003** |
| I/II | 35/74(47.3%) | 3/23(13%) |  |
| III/IV | 39/74(52.7%) | 20/23(87%) |  |
| IPI |  |  | 0.051 |
| 0-1 | 39/74(52.7%) | 6/22(27.3%) |  |
| 2-5 | 35/74(47.3%) | 16/22(72.7%) |  |
| B-symptom |  |  | 0.126 |
| yes | 21/74(28.4%) | 11/23(47.8%) |  |
| no | 53/74(71.6%) | 12/23(52.2%) |  |
| Primary site |  |  | 0.121 |
| Nodal | 53/74(71.6%) | 12/23(52.2%) |  |
| Extranodal | 21/74(28.4%) | 11/23(47.8%) |  |
| LDH>220 IU/L |  |  | 0.609 |
| yes | 31/54(57.4%) | 8/14(57.1%) |  |
| no | 23/54(42.6%) | 6/14(42.9%) |  |
| Response to CHOP |  |  | **0.045** |
| CR+PR | 35/48(72.9%) | 5/13(38.5%) |  |
| SD+PD | 13/48(27.1%) | 8/13(61.5%) |  |

COO cell of origin; GCB germinal center B-cell-like; PS performance status; IPI International Prognostic Index; LDH lactate dehydrogenase; CHOP cyclophosphamide, doxorubicin, vincristine, prednisone; CR complete remission; PR partial remission; SD stable disease; PD progressive disease.
